# Is Brain-Derived Neurotrophic Factor a Metabolic Hormone in Peripheral Tissues?

**DOI:** 10.3390/biology11071063

**Published:** 2022-07-17

**Authors:** Elsie Chit Yu Iu, Chi Bun Chan

**Affiliations:** School of Biological Sciences, The University of Hong Kong, Hong Kong SAR, China; elsiecyiu@connect.hku.hk

**Keywords:** BDNF, metabolism, mitochondria, peripheral tissues

## Abstract

**Simple Summary:**

The activity of brain-derived neurotrophic factor (BDF) in the central nervous system has been well-studied, but its physiological role in other organs has not been clearly defined. This review summarizes the current findings on the functionality of BDNF in various peripheral tissues and discusses several unresolved questions in the field.

**Abstract:**

Brain-derived neurotrophic factor (BDNF) is an important growth factor in the central nervous system. In addition to its well-known activities in promoting neuronal survival, neuron differentiation, and synaptic plasticity, neuronal BDNF also regulates energy homeostasis by modulating the hypothalamus’s hormonal signals. In the past decades, several peripheral tissues, including liver, skeletal muscle, and white adipose tissue, were demonstrated as the active sources of BDNF synthesis in response to different metabolic challenges. Nevertheless, the functions of BDNF in these tissues remain obscure. With the use of tissue-specific Bdnf knockout animals and the availability of non-peptidyl BDNF mimetic, increasing evidence has reported that peripheral tissues-derived BDNF might play a significant role in maintaining systemic metabolism, possibly through the regulation of mitochondrial dynamics in the various tissues. This article reviews the autocrine/paracrine/endocrine functions of BDNF in non-neuronal tissues and discusses the unresolved questions about BDNF’s function.

## 1. Introduction

**Figure 1:**
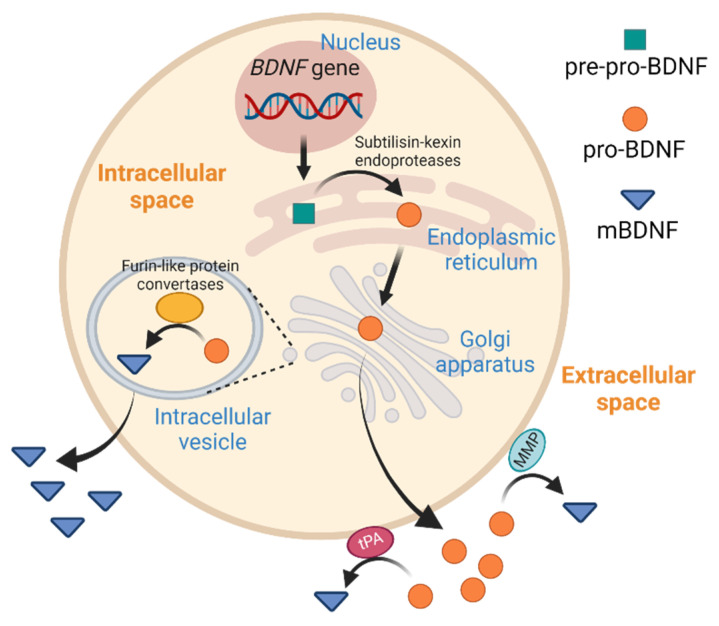
Synthesis of pro-BDNF and mature BDNF (mBDNF). *BDNF* mRNA is translated in the endoplasmic reticulum to form pre-pro-BDNF, which is subsequently cleaved to form pro-BDNF. After being transported into the Golgi apparatus, the pro-BDNF is further converted into mBDNF by furin-like protein convertases. Alternatively, pro-BDNF is exported as a functional hormone or is further processed by the tissue type plasminogen activator (tPA) or matrix metalloproteinase (MMP) to form mBDNF extracellularly.

Together with nerve growth factor (NGF), Neurotrophin-3 (NT-3), and Neurotrophin-4 (NT-4), brain-derived neurotrophic factor (BDNF) is a member of the structurally related neurotrophin family that plays crucial roles in neurological activities, such as neurogenesis during the tissue development, differentiation and survival of neurons, regulation of synaptic plasticity for memory formation, and guidance of tissue–neuron interaction. Mature BDNF (designated as mBDNF in this review) exists as a dimer of two non-covalently linked peptides, which is formed after the intracellular endopeptidase cleavage of pro-BDNF in the endoplasmic reticulum or via the membrane-bound protease like matrix metalloproteases extracellularly (Figure 1) [1]. In addition to serving as the precursor for mBDNF synthesis, pro-BDNF might work as a functional protein via interacting with the p75 neurotrophin receptor (p75NTR)-sortilin complex [2]. In contrast, mBDNF exerts biological functions via binding to the single transmembrane receptor tyrosine kinase, tropomyosin receptor kinase B (TrkB). The binding of mBDNF to TrkB initiates three major signaling cascades: phosphoinositide 3-kinase (PI3K)/protein kinase B (Akt), mitogen-activated protein kinase (MAPK)/extracellular-signal-regulated kinase (ERK), and phospholipase C γ (PLCγ)/cAMP response element-binding protein (CREB) pathways, which upregulate the transcription of pro-survival genes in the brain [3]. The Ca^2+^ influx that follows PLCγ activation also increases the activity of the N-methyl-D-aspartate (NMDA) receptor [3], which contributes to synaptic maturation and memory formation [4]. Because neuronal BDNF plays an important role in long-term potentiation, synaptic plasticity, and neurogenesis, reduced *BDNF* expression is associated with neuronal diseases such as bipolar disorder, Huntington’s disease, Alzheimer’s disease, and Parkinson’s disease [5]. The neurotrophic functions of BDNF have been extensively described, and readers who are interested in this topic are referred to other excellent reviews [6,7,8].

Studies in *Bdnf* and *Ntrk2* (the TrkB gene) knockout mice revealed that mBDNF participates in the regulation of body weight through controlling food intake [9,10]. In the fed state, high serum glucose and leptin levels activate the neurons that express cocaine- and amphetamine-regulated transcript/pro-opiomelanocortin (POMC) in the arcuate nucleus in the hypothalamus to prevent over-eating [11,12]. The anorexigenic activities of POMC-positive neurons depend on the production of 𝛼-melanocyte stimulating hormone (𝛼-MSH) as the neurotransmitter, which activates melanocortin 4 receptor activity of other feeding-control neurons in multiple brain regions, such as the ventromedial hypothalamus (VMH) [13]. Xu et al. have reported that *Bdnf* is an 𝛼-MSH-responsive gene in the neuron of the VMH and ablation of *Bdnf* or *TrkB* in these neurons causes hyperphagia and excessive weight gain in mice [10,14]. Corroborating these findings, mutation of *BDNF* or *NTRK2* in human beings leads to the development of obesity and other related metabolic disorders [15,16]. Interestingly, subcutaneous infusion of mBDNF effectively mitigated glucose homeostasis in obese *db/db* mice after pair-feeding, suggesting that mBDNF is able to manage systemic metabolism independently of its anorexigenic functions [17]. Although it has been proposed that subcutaneously administrated mBDNF regulates the integral metabolism via the neuronal output to various tissues, mBDNF might also act directly on peripheral tissues to orchestrate their metabolic functions. Indeed, mBDNF and TrkB proteins can be found in key organs for metabolic controls, including the liver, pancreas, skeletal muscles (SkM), and white adipose tissues (WAT), but their authentic functions in these peripheral tissues are still ambiguous [18,19]. With the help of genetic engineering that confines the overexpression or ablation of *Bdnf* or *Ntrk2* in a single tissue, it is now clear that BDNF is involved in numerous tissue-specific and systemic metabolic activities. This review will outline the actions of tissue-specific BDNF in local and systemic metabolism regulation and discuss several key issues to fully elucidate the functional spectrum of peripheral tissue-derived BDNF.

## 2. BDNF in Liver

**Figure 2:**
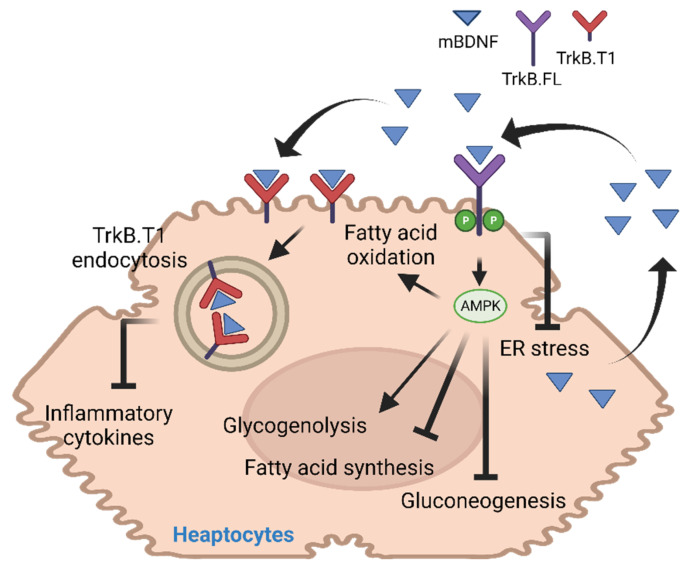
Functional activities of mBDNF in the liver.

The liver is a vital organ for maintaining metabolic homeostasis. During the fasting state, high glucagon and low insulin levels increase hepatic glucose production to stabilize the blood glucose concentration [20]. On the other hand, excessive glucose in dietary sources is converted into triacylglycerides (TAG) via de novo lipogenesis in the liver, which is delivered to other tissues via very-low-density lipoproteins. Disruption of these regulatory processes leads to the development of metabolic diseases, including hyperlipidemia, hyperglycemia, and hepatic steatosis [21]. Although regulation of hepatic metabolism depends mainly on the signal from the pancreas via the production of insulin and glucagon, subcutaneous injection of mBDNF also potentiates insulin-induced PI3K activity in the liver [22]. However, it is not clear if mBDNF directly activates its receptor in hepatocytes to potentiate insulin sensitivity, because *Ntrk2* expression in hepatocytes is very low [23]. Moreover, the observation that intracerebroventricular infusion of mBDNF suppresses hepatic glucose production, possibly acting via the vagus nerve, further questions the direct action of mBDNF on hepatocytes [24]. When *Bdnf* was overexpressed in the mouse liver with non-alcoholic steatohepatitis by AAV-mediated gene delivery, however, the animals displayed lower hepatic damage, reduced inflammatory gene expression, and improved fibrosis and steatosis, indicating that the hepatocyte-synthesized BDNF has a local protective function to the liver against metabolic challenges [25]. Studies in cultured mouse hepatocyte cell lines AML12 provided further proof that mBDNF is a direct stimulator of fatty acid oxidation (FAO) and glycogenesis but a suppressor of the hepatic fatty acid (FA) synthesis and gluconeogenesis [26]. Furthermore, BDNF deficiency in the heterozygous *Bdnf* knockout mice (BDNF+/−) sensitizes their hepatocytes to ER stress-induced cell death, which is a common consequence seen in the obese tissues [27]. Hence, the FA-induced *Bdnf* expression in the livers of mice after high fat diet (HFD) feeding might prevent the ectopic lipid accumulation and the related adverse metabolic consequences [26,28]. Mechanistically, mBDNF destabilizes the TrkB isoform, TrkB.T1, in hepatocytes to protect the cells from lipotoxicity [25]. Generated by alternative splicing of the *Ntrk2* gene, TrkB.T1 is a truncated isoform of the full-length TrkB (Trkb.FL) without the kinase and C-terminal domains [29]. Because *TrkB.T1* expression was increased in the mouse liver after HFD feeding, which potentiated the TNFα-induced cell damage and inflammatory response through a ligand-independent mechanism, the high mBDNF production in the HFD-fed mice prevents further damage by reducing the cellular content of TrkB.T1 [25]. Nevertheless, an opposing view on BDNF’s protective action on the liver has also been proposed. For instance, higher hepatic content of mBDNF is detected in patients with major depressive disorder, schizophrenia, and bipolar disorder, and the authors proposed that hepatic mBDNF might contribute to the high incidence of liver disease in these psychiatric diseases [30]. In support of this notion, hepatocyte-specific *Bdnf* knockout mice displayed reduced liver damage, alleviated hepatic steatosis, and augmented FAO in the liver when the knockout animals were fed with HFD, suggesting *Bdnf* expression might not be beneficial to the liver metabolism [31]. It remains to be determined why opposite functions are observed in mice after HFD feeding, and more functional characterization of liver-specific *Bdnf* knockout mice is definitely needed to clarify the metabolic role of BDNF in the liver (Figure 2).

## 3. BDNF in Adipose Tissue

WAT is the chief energy reservoir of mammals that catabolizes the stored TAG via lipolysis to fuel other peripheral tissues during energy scarcity. Since the discovery of leptin and its activities in the central nervous system (CNS) to control the systemic energy metabolism, however, the functionality of WAT has been changed from a simple and inert energy depot to an organ that actively shapes whole-body metabolism. It is now widely accepted that WAT is a metabolic tissue for lipid storage, adipokines secretion, and insulin sensitivity maintenance [32]. A significant amount of BDNF could be detected in WAT [19,33], whose expression was induced by streptozotocin injection [34] or HFD feeding [35]. Because WAT is a heterogeneous tissue that contains multiple cell types, including mature adipocytes, hematopoietic lineage of immune cells, preadipocytes, vascular endothelial cells, and pericytes [36], the elevated *Bdnf* expression in WAT might not necessarily occur in adipocytes. Studies in various cell type-specific *Bdnf* knockout mice have revealed a complex response of BDNF in WAT. Firstly, *Bdnf* expression in WAT was not abolished in adipocyte-specific knockout mice, suggesting most *Bdnf* expression occurs in the cells of the heterogeneous stromal vascular fraction (SVF) of WAT [35]. In another study performed in the myeloid lineage-specific *Bdnf* knockout mice, only a mild reduction of BDNF in the SVF was found, which excludes the possibility that adipose immune cells are the primary source of BDNF production in the tissue [37]. Presumably, the adipose progenitor cells might be the major cell types of BDNF synthesis in WAT. Indeed, human preadipocytes have a high expression of *Bdnf*, which is significantly reduced during adipogenesis [33]. Because inhibiting *Bdnf* expression in pre-adipocytes led to a mild reduction in adipocyte differentiation, it is suggested that BDNF is only critical to the commitment of progenitor cells to form adipogenic cell lineage but not the maturation of preadipocytes [33]. However, later studies showed that treatment of 3T3-L1 preadipocyte with 7,8-dihydroxyflavone (7,8-DHF), a bioavailable non-peptidyl BDNF mimetic [38], reduced adipocyte differentiation via inhibiting the expression of key adipogenic transcription factors such as CCAAT/enhancer binding protein α and peroxisome proliferator-activated receptor γ [39,40], suggesting BDNF is an inhibitory factor to the formation of new adipocytes.

Recently, it was proposed that the BDNF production in progenitor cells of WAT is detrimental to the tissue’s metabolic health during aging. Using the inducible adipocyte progenitor-specific *Bdnf* knockout (BDNF^Pdgfra^ KO) mice, Song et al. demonstrated that abolishing the production of BDNF in adipocyte progenitor cells prevents the aging-induced inflammation and glucose intolerance [41]. Because in vitro assays showed that pro-BDNF promoted adipocyte apoptosis and reduced the cellular mitochondria content, the authors concluded that an excessive pro-BDNF production in the adipose progenitor cells of aged animals triggered the death of adipocytes, causing the infiltration of immune cells and deterioration of the metabolic fitness. Nevertheless, the study did not include an assessment of the production and functional activity of mBDNF in the aged WAT, and it remains unknown if the elevated pro-BDNF production is an isoform-specific event or an overall increase of *BDNF* transcription that enhances the mBDNF synthesis as well.

Although the above studies suggest that mature adipocytes might not produce mBDNF, they do respond to both pro-BDNF and mBDNF stimulations as a significant amount of TrkB and p75NTR could be found in WAT [35,42]. Moreover, more mitochondrial fission and browning were detected in the differentiated 3T3-L1 adipocytes after BDNF stimulation [43]. It is possible that the non-adipose cells in WAT produce BDNF paracrine to modulate the metabolic activities of their adjacent adipocytes. While there are no direct studies on BDNF’s metabolic modulation in adipocytes, the TrkB-BDNF signaling in the adipocyte is important to food intake regulation as adipocyte-specific TrkB knockout (Adipoq-TrkB CKO) mice fed a high-fat/high-sucrose (HFHS) diet displayed hypophagia [35], indicating that the TrkB in adipocyte is responsible for generating a stimulatory afferent input to the CNS for initiating feeding behavior. Because *Ntrk2* expression in WAT is diminished in diet-induced obese mice [35], the TrkB in WAT might represent an adiposity signal that suppresses further food intake when the animals consume energy-dense food.

**Figure 3:**
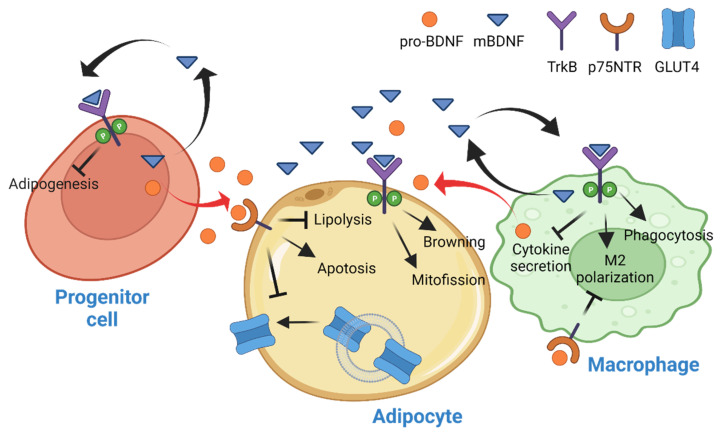
Functional activities of mBDNF and pro-BDNF in the white adipose tissue. The mBDNF and pro-BDNF are mainly synthesized by the progenitor cells and macrophages, which act on the mature adipocyte to modulate its metabolism. The BDNFs might also act as an autocrine to modulate the differentiation of pre-adipocytes and the transformation of macrophages.

Macrophages and their monocyte precursors comprise the highest fraction of immune cells in adipose tissues [44]. During obesity development, the number of pro-inflammatory M1 adipose tissue macrophages (ATM), but not the resolving M2 ATM, is significantly increased in WAT, contributing to a chronic state of tissue inflammation [45]. These macrophages express bioactive BDNFs (both mature and pro-BDNF) and TrkB [46,47,48,49]. Several studies have demonstrated that mBDNF stimulation promoted the activation of M1 to M2 macrophage transformation, suppressed inflammatory cytokine secretion, and triggered the migration of macrophages towards the damage site [50,51,52,53]. Since the hypertrophic adipocytes in obese animals produce tumor necrosis factor α (TNFα) and interleukin 6 (IL-6), which are stimulators of BDNF synthesis in monocytes [54,55], it is tempting to hypothesize that BDNF production in ATM is a protective mechanism to promote the formation of M2 macrophages in response to the HFD feeding. In line with this hypothesis, myeloid-specific *Bdnf* knockout mice, which have no *Bdnf* expression in their monocytes, mature macrophages, and granulocytes, displayed lower energy expenditure and exacerbated adiposity when they were fed with HFD [37]. Because the authors have not examined the concentration of type 1 cytokines that cause metabolic dysfunctions in the HFD-fed KO mice, whether the immune cells-derived BDNF contributes to the inflammatory responses that are commonly seen in WAT of obese animals remains unanswered. Instead, Blaszkiewicz et al. showed that the myeloid-derived BDNF is essential for maintaining sympathetic innervation in WAT [37], which resembles the axon guidance role of BDNF in the CNS [56]. In contrast to the stimulatory activities of mBDNF, pro-BDNF has an inhibitory effect on macrophage activation and migration, but the metabolic outcomes of enhanced pro-BDNF production on ATM’s behavior have not been explored [49]. Instead, the studies on the pro-BDNF receptor, p75NTR, in adipocytes provide hints on the metabolic functions of pro-BDNF in adipose tissue. When the p75NTR gene (*Ngfr*) was ablated in adipocytes, lipolysis and membrane translocation of glucose transporter (GLUT4) were enhanced [42,57]. Hence, the adipocyte-specific *Ngfr* knockout mice are resistant to HFD-induced adiposity, hepatic steatosis, and insulin resistance. These protective effects were not observed in the skeletal muscle *Ngfr* knockout mice, suggesting that WAT is the primary site of metabolic action for p75NTR [42]. On the other hand, *Ngfr* overexpression in cultured adipocytes attenuated lipolysis and lipid oxidation by suppressing the activity of protein kinase A. Hence, the elevated *Ngfr* expression exclusively in WAT of HFD-fed mice provides an additional mechanism to account for the dysregulated lipid metabolism in the tissue [58] (Figure 3).

In short, BDNF in WAT is mostly synthesized by the non-adipose cells to modulate the cellular activity of mature adipocytes.

## 4. BDNF in Skeletal Muscle

*Bdnf* mRNA can be detected in the soleus, tibialis anterior (TA), extensor digitorum longus (EDL), gastrocnemius, and diagram muscles [59,60,61]. Within the soleus muscle, both slow- and fast-type myofibers express *Bdnf* [62], but type II glycolytic myofibers contain higher expression of *Bdnf* than type I oxidative myofibers [63]. In humans and rats, increased *BDNF* expression and protein content in Skm are observed following a single running session or regular treadmill trainings [64,65]. Because electrical stimulation is a potent inducer of BDNF secretion in cultured muscle, it is believed that the myofiber contraction is the primary driving factor of BDNF production during exercise, but the functional significance of elevated mBDNF content in Skm after exercise remains obscure [66,67,68]. In pioneer studies to determine the mBDNF’s function in cultured muscle cells, it has been shown that mBDNF was able to stimulate FAO via AMP-activated protein kinase (AMPK) activation [60,66]. A similar observation of AMPK-induced FAO was found in C2C12 after TrkB stimulation by 7,8-DHF [69]. Our recent report further demonstrated that BDNF activated AMPK in the muscle cells via triggering an intracellular Ca^2+^ surge, which acted through the calmodulin K kinase 2 (CamKK2) to induce AMPK phosphorylation [28]. AMPK is an imperative metabolic sensor that balances the energy metabolism, whose activity is provoked under an energy-deficient state. In the cells that are experiencing energy deficit, AMPK activation inhibits the anabolic process in order to reduce ATP consumption and promotes the catabolic process, which generates ATP [70]. Hence, it is suggested that BDNF in muscle is mainly responsible for the elevation of FAO to meet the energy demand of Skm during exercise by provoking the activity of AMPK [71]. However, studies in transgenic mice that express a kinase-dead AMPKα2 in Skm [72] or in inducible muscle-specific *Prkaa1* and *Prkaa2* (AMPK α1 and α2 subunit genes) double-knockout mice demonstrated that the contraction-induced FAO was not impaired in their muscle [73], suggesting AMPK activation is dispensable for FAO during exercise. Instead, O’Neill et al. reported that AMPK in Skm is needed for the glucose uptake stimulated by contraction [74]. The role of BDNF in muscle performance during exercise is also unascertained as contradictory results have been reported in studies using muscle-specific *Bdnf* knockout (MBKO) mice. While we found that the total daily locomotion, exercise endurance, and muscle strength were weakened in mice without BDNF in their muscle, Delezie et al. observed that MBKO mice had greater resistance to contraction-induced fatigue, although they also found the mice displayed lower daily locomotion [60,63]. Hence, the relationship between BDNF-AMPK signaling and exercise-provoked muscle responses needs further verification.

FAO in muscle is also increased during fasting [75]. While glucose uptake, glycolysis, and pyruvate oxidation are favored in the postprandial period when glucose consumption is high, FAO is suppressed in Skm, WAT, and liver to ensure that tissue exposure to hyperglycemia is minimized. On the other hand, the inhibited FAO is unlocked by the action of AMPK via phosphorylating acetyl Co-A carboxylase (ACC) directly, making FA the primary energy source during fasting [76,77]. This “fuel selection (or metabolic flexibility)” is a pivotal response to spare glucose for organs, such as the brain, that utilize glucose as their sole energy source [78,79]. At the molecular level, metabolic flexibility relies on the configuration of signaling pathways that manage nutrient sensing, uptake, transport, storage, and utilization. In cultured C2C12 myotubes, BDNF secretion was provoked by the biochemical factors of fasting, including glucose depletion, amino acid restriction, and β-hydroxybutyrate (βHB) stimulation [28,60,80,81]. We and others have also shown that *Bdnf* expression in Skm is increased during fasting, suggesting mBDNF might be involved in regulating metabolism to move through the fed-fast cycle [60,81]. Giacco et al. further demonstrated that fasting-induced *Bdnf* expression is associated with elevated phosphorylation of cAMP-responsive element-binding protein (CREB), TrkB, and AMPK in the skeletal muscle [81]. Despite the elevated *Bdnf* expression in Skm, the activity of Akt, a major downstream effector of BDNF-TrkB signaling in neurons [82], is downregulated during fasting [81]. Possibly, BDNF might provoke tissue-specific cascades or other fasting-induced responses overwhelm the stimulatory effect of BDNF in muscle during fasting, which requires further exploration. In any case, MBKO mice could not handle the increased FA influx to Skm during fasting because of the impaired FAO ability, leading to the ectopic accumulation of lipids. Eventually, the animals develop lipotoxicity-induced insulin resistance [60]. On the other hand, excessive energy supply such as HFD feeding suppresses *Bdnf* expression in the Skm, leading to insufficient AMPK phosphorylation, exaggerated accumulation of lipids, and severe diet-induced insulin resistance [28]. These findings suggest that the BDNF-AMPK cascade in Skm is a homeostatic signaling to cope with nutrient availability.

In addition to lipid metabolism, AMPK is crucial to mitochondrial remodeling and homeostasis via controlling the mitochondrial biogenesis, regulating the shape of the mitochondrial network, and clearing the defective mitochondria [83].

Mitochondrial biogenesis occurs when a cell experiences a high energy demand. The increase of mitochondrial content requires the transcription of genes encoded in the nuclear and mitochondrial genomes. Gain- or loss-of-function studies show that AMPK activity positively correlates with the mitochondrial number in Skm [84,85]. Mechanistically, AMPK promotes mitochondrial biogenesis via modulating the activity of peroxisome proliferator-activated receptor γ co-activator 1 α (PGC-1α), which is an inducer of transcriptional coactivation of TRF-1 (Nuclear Respiratory Factor 1) and TFAM (Transcription Factor A, Mitochondrial) [86]. Through direct phosphorylation and expression control, AMPK promotes the activity of PGC-1α to increase the number of mitochondria in Skm [87]. Several studies have shown that BDNF or 7,8-DHF stimulation increased the mitochondrial content and cellular respiration in Skm via the AMPK-PGC-1α pathway in mice [28,60,88,89]. Consequently, BDNF administration or 7,8-DHF consumption effectively reduces the body weight gain of mice under HFD feeding and ameliorates the locomotion after myocardial infarction [88,89]. Stimulation of mitochondrial biogenesis is possibly a universal function of BDNF, as this activity could also be detected during neuronal dendritogenesis [90]. Nevertheless, ERK and CREB, but not AMPK, are responsible for BDNF-promoted PGC-1α and mitochondrial biogenesis in neurons [90]. Because BDNF is able to induce CREB phosphorylation, it is possible that the BDNF-elevated PGC-1α in myotubes is also CREB-dependent [60]. It is also interesting to find that AMPK-PGC-1α signaling and mitochondrial content were only reduced in the MBKO mice during fasting but not in the fed status, which reinforces the physiological role of BDNF as a stress-induced myokine to cope with the energy demand [60]. Presumably, the drop of extracellular glucose and the increase of βHB content in muscle [81] during fasting promote the BDNF production in muscle fibers, which activates AMPK-PGC-1α pathways to facilitate mitochondrial biogenesis and the glycolysis-to-FAO shift.

Defective AMPK signaling in the MBKO muscle not only decreases the synthesis of new mitochondria but also hinders the clearance of the faulty mitochondria. In energy-deficient events, such as exercise and fasting, a large amount of reactive oxygen species (ROS) is generated by the electron transport chain (ETC) complex I and III, which imposes significant damage to the mitochondrial proteins [91]. Interestingly, increased glucose and FA intake in obesity also elevates ROS production, contributing to mitochondrial dysfunction [92]. Mitochondrial fragmentation (mitofission) is facilitated in these conditions to segregate the damaged organelle portion for selective degradation by mitophagy [93]. AMPK is an upstream regulator of the process, as ablating *Prkaa1* and *Prkaa2* expression results in suppressed mitofission in a variety of cell types [94,95]. While activated AMPK triggers mitofission through phosphorylating the mitochondrial fission factor (MFF) directly, which is the pre-requisite for dynamin-related protein 1 (DRP1) recruitment to induce mitochondrial division [94], it also phosphorylates Unc-5 like activating kinase 1 (ULK1) to promote phagophore formation for non-selective autophagy [96]. The activated ULK1 then induces rapid phosphorylation on the ubiquitin ligase Parkin to prepare its mitochondrial retention [97]. Moreover, AMPK phosphorylates and promotes the accumulation of PTEN-induced kinase 1 (PINK1), a critical inducer of mitophagy [98], at the mitochondrial membrane surface. PINK1 at the mitochondrial membrane phosphorylates the ubiquitin ligase Parkin, which is a critical post-translational modification for the mitochondrial localization and ligase activity of Parkin towards mitochondrial membrane proteins such as voltage-dependent anion channel (VDAC) [99]. Adaptors, including p62, optineurin (OPTN), and nuclear dot protein 52 kDa (NDP52), then recognize the polyubiquitinated mitochondrial proteins and bridge them with the LC3 on phagophore for autophagosome formation [100]. When AMPK is inhibited genetically or pharmacologically, the mitofission and removal of stressed mitochondrial are hampered [101,102]. As an upstream activator of AMPK, stimulation of C2C12 myotubes with mBDNF promotes mitophagy in an AMPK-dependent manner [28]. Interestingly, Skm-derived mBDNF is dispensable for basal mitochondrial dynamics, but is critical to mitophagy initiation when the cells are under metabolic stress, such as the palmitic acid challenge [28]. Hence, the clearance of damaged mitochondria is diminished in the muscle of MBKO mice, leading to the accumulation of dysfunctional mitochondria for efficient FAO, severe insulin resistance, and obesity [28]. It is noteworthy that BDNF- or 7,8-DHF-regulated mitophagy could also be detected in cultured cardiomyocytes, adipocytes, retinal ganglion, and vascular endothelial cells, but whether AMPK in these cells is involved in the process remains to be determined [43,103,104,105]

In addition to muscle [88,106], BDNF has a significant role in regulating the mitochondrial respiration in other tissues. For instance, mBDNF or 7,8-DHF augments the mitochondrial respiration in neuron preparation [107], injured cortical neurons, exfoliated deciduous stem cells-differentiated dopaminergic neurons [108], retinal ganglion cells [105], neuroblastoma [109], cultured cardiomyocytes [110], and placenta trophoblasts [111]. The detailed mechanism of how BDNF modulates mitochondrial respiration remains largely unknown, but the localization of TrkB.FL and TrkB.T1 on mitochondrial membranes provides a possibility that BDNF might act directly on the organelle to regulate its activity [112].

**Figure 4:**
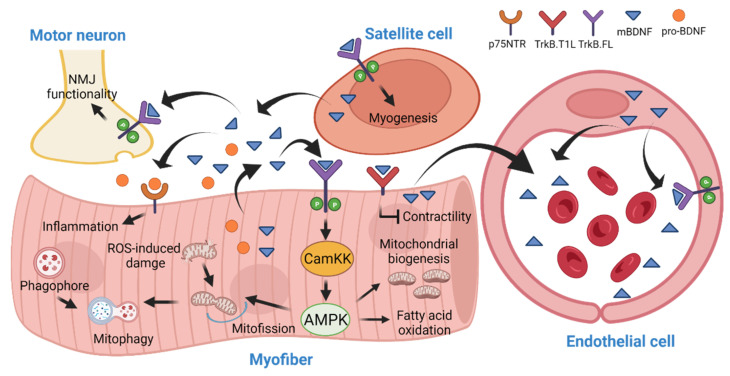
Functional activities of BDNF in skeletal muscle (Skm). In Skm, mBDNF is produced by the myofiber, satellite cells, and blood vessel endothelial cells. It acts in an autocrine and paracrine manner to modulate the mitochondrial dynamics and lipid metabolism in myofibers, control the myogenesis, and maintain the functions of the neuromuscular junction. The myofiber-generated BDNF is also secreted into circulation to regulate the functions of the pancreas.

BDNF is also involved in muscle development (myogenesis) and regeneration. These processes require a series of clonal expansion, differentiation, and fusion of muscle cells. During myogenesis, the small multipotent satellite cells (SCs) will be activated and exit the cell cycle to form myoblasts, which align spatially into chains and fuse into the multinucleated myotubes. Muscle regeneration also requires the recruitment SCs to the site of injury, where they are differentiated and fused to form multinucleated myotubes [113]. A high expression of *Bdnf* was detected in cultured myoblasts, which was downregulated during myogenic differentiation and fusion [61,114,115]. Reduced *Bdnf* expression can also be detected in the developing muscle of mice [61]. Seidl et al. hypothesized that the presence of myoblast-derived neurotrophin is essential to support the myogenic cell migration. When these cells have reached their ultimate position, terminal differentiation is initiated by the downregulation of neurotrophin synthesis. The cessation of neurotrophin production from the mature muscle cells also provides a cue to proper innervation by eliminating the unnecessary motoneuron synapse [114]. This hypothesis is supported by the observation that chronic mBDNF stimulation decreased the synaptic maturation of the neuromuscular junction (NMJ) in the Xenopus neuron-muscle co-culture [116]. However, contradictory findings have also been reported that mBDNF promotes the structural and functional maturation of neuromuscular synapses via TrkB.FL activation [117]. Garcia et al. further proposed that a minimal synthesis of BDNF from neonatal muscle is indispensable, as it serves as a retrograde modulator to upregulate neurotransmission in all synaptic contacts, regardless of the level of axonal maturation [118]. In alignment with this hypothesis, disrupting the activity of TrkB.FL or overexpressing the TrkB.T1 on the postsynaptic muscle fiber resulted in the disassembly of acetylcholine receptor cluster at the motor endplate [119]. Because the functional ability, apposition, and integrity of the motor endplate were not altered in the MBKO mice [63], the BDNF that is necessary for maintaining the postsynaptic functions might possibly come from the motor neuron or other cell types, such as Schwann cells or satellite cells. Indeed, *Bdnf* expression was found in “active (Pax7+/MyoD+)” SCs during early differentiation [120], and BDNF stimulation induced a significant increase in myoblast proliferation [120]. Because BDNF content in Skm increases after exercise-induced injury, it is believed that *Bdnf* expression is important to myogenesis initiation during muscle regeneration [120,121]. Although *Bdnf* ablation in SCs did not compromise their differentiation into the myogenic lineage in mice, the expression of differentiation markers of later steps of myotube differentiation was significantly reduced, resulting in a delay in the early regeneration of Skm after cardiotoxin-induced injury [122] (Figure 4).

Ultimately, BDNF in muscle is not only synthesized by myofibers, but other cells, such as satellite cells and blood vessel endothelial cells, also play a significant role. In addition to the autocrine/paracrine activities to regulate the metabolism of myofibers and muscle regeneration, muscle-derived BDNF serves as a hormone to communicate with other tissues such as the pancreas for maintaining systemic glucose and lipid homeostasis (to be discussed in the next section).

## 5. Unresolved Questions

In comparison with the studies performed in CNS, our understanding of BDNF’s activity in peripheral tissues is still rudimentary. Studies on BDNF’s function and expression regulation in various non-CNS tissues are fragmented, and inconsistent results are frequently reported, possibly due to the distinct and opposite functions of BDNF isoforms, especially in gene knockout studies where both pro-BDNF and mBDNF are depleted. For instance, ablating the *Bdnf* gene in the Skm mitigates the denervation-induced muscle atrophy but overexpressing *Bdnf* also has an ameliorating effect on the defective muscle function in neuromuscular disease [123,124]. Moreover, the lack of precise tools significantly impedes the result interpretation and conclusion to be made. An obvious example is the non-specificity of antibodies that recognize BDNF and TrkB. Because most commercially available antibodies show a certain degree of cross-reactivity with other proteins, it is difficult to accurately determine the cell types that express BDNF and TrkB in tissue or serum. This technical issue can be solved using the knock-in animals with a highly specific tag fused to the BDNF protein or immunoprecipitation as demonstrated by Fulgenzi et al. [68]. Nevertheless, the pathological features in the muscle- or liver-specific *Bdnf* knockout mice have proved that peripheral tissue-generated BDNF (either mBDNF or pro-BDNF) is equally important to the CNS-derived BDNF in maintaining metabolic homeostasis. Thus, enhancing the BDNF signaling has a beneficial effect on the overall metabolism, particularly in preventing or treating metabolic and neurological disorders [69,88,125].

To better understand the functions of BDNF outside the CNS, a foremost important question to be solved is the source that contributes to the change of circulating BDNF in various physiological and pathological conditions [121,126,127,128]. Because the concentration of BDNF in platelet-poor plasma is low, it is proposed that megakaryocyte/platelet is the origin of BDNF in blood. However, the increased BDNF level in plasma after exercise suggests that circulating BDNF can be originated from other tissues [129]. By comparing the circulating BDNF concentrations between the radial artery and jugular vein, it is concluded that the brain contributes ~70% of BDNF in blood during resting and exercise [130,131]. Nevertheless, there are arguments against the contribution of brain to the circulating BDNF level because in conditions such as stroke and exercise, where the concentration of BDNF in the brain is increased, there is no change in the blood BDNF content [132]. Although Mathew et al. demonstrated that BDNF is an autocrine or paracrine in muscle to regulate the local tissue function because over-expressing *Bdnf* transiently in skeletal muscle did not change the BDNF concentration in the blood [66], a study using the knock-in mice with a V5 tag in the *Bdnf* locus showed that muscle-derived BDNF could be secreted into the circulation [68]. Our findings that the amount of circulation BDNF is significantly reduced in the MBKO mice during fasting support this notion [60]. Presumably, the electroporation-mediated *Bdnf* overexpression in hindlimb muscle as performed by Mathew et al. might not produce a sufficient amount of BDNF to be detected in the circulation. A recent report further argues that the endothelial cells but not the myofibers are the major production site of BDNF in Skm, which may account for the elevation of blood BDNF levels in response to physical exercise [62]. It is also possible that the BDNF in blood is a combinatory secretion from megakaryocytes, endothelial cells, lymphocytes, monocytes, and interstitial fluid from different peripheral tissues, and CNS. In spite of these arguments on the source of BDNF in muscle, the release of Skm-derived BDNF into circulation is undoubtful, which implies that BDNF might function as a hormone to communicate with other tissues. Indeed, Fulgenzi et al. have demonstrated that the BDNF from Skm is responsible for inducing insulin secretion from the pancreatic β-cells, which might represent an inter-organ communication for normalizing hyperglycemia following exercise [68,133]. It would be interesting to investigate in future if BDNF production in other peripheral tissues, such as the liver, would also serve as an endocrine to orchestrate the metabolism or functions in different tissues.

It is also important to determine the dominant receptor of BDNF to exert its metabolic functions in different tissues. The dogma that BDNF only relies on TrkB.FL to initiate the biological activities has been changed since the discovery that TrkB.T1 could transduce intracellular signals by provoking intracellular calcium ([Ca^2+^]_i_) release [134]. This finding also overturns the idea that TkrkB.T1 only acts as a dominant-negative inhibitor of TrkB-T1 or limits the availability of BDNF for TrkB.FL binding [135,136]. However, the function of TrkB.T1 in non-nervous tissues is less studied. In tissues where [Ca^2+^]_i_ is crucial in their cellular function, such as cardiomyocytes and the pancreatic β-cells, deleting *TrkB.T1* abolished the action of BDNF, which impedes heart contraction and insulin secretion [68,137]. Because TrkB.FL is hardly detectable in these tissues, the TrkB.T1 is assumed to be the predominant receptor for BDNF to regulate calcium homeostasis. In contrast, TrkB.T1 seems to work as a negative inhibitor of TrkB.Fl in the Skm as depleting the *TrkB.T1* gene results in a greater Ca^2+^ flux between the cytoplasm and sarcoplasmic reticulum to increase contractility [138]. The differential expression of *TrkB.FL* and *TrkB.T1* as well as their functional interaction in various tissues might represent an additional regulatory mechanism for BDNF response in peripheral tissues. Hence, it would be necessary to consider the role of TrkB.T1 in studying the BDNF’s function in the future.

Last but not least, peripheral tissues-produced mBDNF might act on the CNS to modulate cognitive functions. When considering together that mBDNF is a well-recognized myokine whose expression is increased after exercise, muscle-derived mBDNF is secreted into the circulation [68]. Although Pardridge et al. demonstrated that BDNF in blood was rapidly degraded and no mBDNF transcytosis through the blood–brain barrier (BBB) in rat was observed [139], later studies showed that blood-borne mBDNF can enter the CNS by a rapid, saturable transport system of the BBB [140,141], mBDNF concentration in blood is reduced in psychiatric and neurological disorders [142], and physical exercise is an effective means to alleviate the mental dysfunction in a lot of psychiatric diseases [143]. Thus, it is tempting to hypothesize that peripheral mBDNF is a beneficial “exerkine” to improve the mental health. The muscle BDNF–brain cross-talk has been partially validated, as adeno-associated virus-mediated overexpression of pro-BDNF in skeletal muscle reduced the dendritic length and density in the brain, leading to the development of depressive behavior [144]. Thus, it would be interesting to study if muscle-derived mBDNF would have a beneficial effect on psychological disorders.

## Data Availability

Not applicable.
